# Optimizing ovulation synchronization in Holstein heifers: implementing a half-dose GnRH analogue (Buserelin Acetate) in a nanofabricated form within the ovsynch protocol to enhance the ovarian activity and reproductive performance outcomes

**DOI:** 10.3389/fvets.2026.1874230

**Published:** 2026-07-07

**Authors:** Eman M. Hassanein, Ottó Szenci, Réka Angi, Ildikó Lipathy, Attila Sánta, Márton Vajna, László Péter, Noémi Anna Niczinger, Nikolett Kállai, István Antal, György T. Balogh, Zoltán Szelényi

**Affiliations:** 1 Department of Obstetrics and Food Animal Medicine Clinic, University of Veterinary Medicine Budapest, Budapest, Hungary; 2 Department of Animal and Fish Production, Faculty of Agriculture, Alexandria University, Alexandria, Egypt; 3 Department of Pharmaceutical Chemistry, Semmelweis University, Budapest, Hungary; 4 Center for Pharmacology and Drug Research and Development, Semmelweis University, Budapest, Hungary; 5 RougeVet Veterinary Practice, Alsónémedi, Hungary; 6 University Pharmacy, Department of Pharmacy Administration, Semmelweis University, Budapest, Hungary; 7 HUN-REN Wigner Research Centre for Physics, Budapest, Hungary; 8 Department of Pharmaceutics, Semmelweis University, Budapest, Hungary; 9 Department of Chemical and Environmental Process Engineering, Faculty of Chemical Technology and Biotechnology, Budapest University of Technology and Economics, Budapest, Hungary

**Keywords:** chitosan nanoparticles, drug delivery, estrus induction, first conception, follicular dynamics, GnRH analogue, luteal function, pregnancy loss

## Abstract

**Introduction:**

The objective of this study was to evaluate the efficacy of a nanofabricated formulation of gonadotropin-releasing hormone analogue, buserelin acetate (GnRH-BA), conjugated with chitosan nanoparticles (CH-NPs) as a nanocarrier. The resulting nano-GnRH-BA formulation was incorporated into the Ovsynch (OVS) protocol at a 50% reduced dose to improve ovulation synchronization and reproductive performance in dairy heifers.

**Methods:**

CH-NPs and nano-GnRH-BA were synthesized via the ionic gelation method. Key characteristics, including particle shape, size, Polydispersity Index (PdI), and drug-loading efficiency (DLE%), were evaluated. A total of 210 Holstein-Friesian heifers were assigned to either a standard OVS (*n* = 82) or nano-OVS (*n* = 128) protocol. Ovarian dynamics were monitored on days 0 (baseline), 9 (before the second GnRH administration), and 27 post-inseminations (post-AI) during the protocol. Reproductive parameters, including estrus induction rate (EIR), first conception rate (FCR), and pregnancy loss (PL), were also recorded. Additionally, the association between ovarian activity and FCR was analyzed.

**Results:**

Baseline ovarian activity (day 0) was similar between groups. By day 9, heifers subjected to nano-OVS exhibited improved follicular development, with larger preovulatory follicles (18.9 ± 1.4 mm) than those subjected to the standard OVS group (16.8 ± 1.3 mm; *P* = 0.007). On day 27 post-AI, larger corpora lutea were observed in the nano-OVS group compared with the standard OVS group (21.3 ± 1.3 mm vs. 18.3 ± 1.4 mm, respectively; *P* = 0.04). These findings were associated with higher EIR (78.6%, *P* = 0.03) and FCR (47.7%, *P* = 0.32), and lower PL (13.1%, *P* = 0.01) compared to the standard protocol (65.9%, 47.7%, and 21.2%, respectively). Correlation analysis exhibited different physiological patterns between groups.

**Discussion:**

Nanofabrication of GnRH may enhance hormone stability and enable sustained release, thereby improving ovulation synchronization and luteal development.

**Conclusion:**

Overall, the nano-GnRH-BA formulation within the OVS protocol resulted in a 50% reduction without compromising fertility, while also improving ovarian function and reducing PL in dairy heifers. This approach may represent a promising alternative to conventional synchronization strategies.

## 1 Introduction

Profitable dairy production system based mainly on efficient reproductive management, particularly in breeds with high genetic merit. In dairy heifers, precise control of ovarian activity is essential for timely breeding, preserving the replacement process, and maximizing profitability (1). Although traditional estrus detection (ED) can be effective under ideal management, it is labor-intensive and often associated with reduced fertility under commercial conditions, and is expected to yield lower net economic value than synchronization protocols (2, 3). Therefore, timed artificial insemination (TAI)-based synchronization protocols have become widely applied in dairy herds (3).

The first described synchronization protocol was the Ovsynch protocol (OVS), which was recognized as a common reproductive management regimen (4). OVS is composed of an initial gonadotropin-releasing hormone (GnRH) administration to trigger ovulation or luteinization of the dominant follicle (DF) and initiate a new follicular wave, followed by prostaglandin F_2α_ (PGF_2α_) administration to induce luteolysis. In addition, a second GnRH administration 48 h later was used to synchronize ovulation before TAI (5, 6).

Although the OVS was initially validated in dairy cows and has been shown to regulate ovarian dynamics and improve fertility outcomes without the need for ED, its efficiency remains suboptimal in heifers due to physiological constraints that limit responsiveness to the protocol (7, 8). Previous studies have confirmed the reduced synchronization efficiency and decreased pregnancy outcomes following OVS application in heifers (5, 9–11), with an approximate pregnancy rate of 35% compared with over 70% in ED-based inseminated heifers (5, 9). These lower responses can be attributed to differences in follicular dynamics between heifers and lactating cows (12). Heifers exhibit rapid follicular growth, a higher prevalence of three-wave cycles, and shorter DF lifespan, all of which may compromise the synchronization efficiency (1, 13). As a result, GnRH administration at a random or suboptimal stage of the estrous cycle may lead to a reduced ovulatory response and impaired synchronization efficiency (14). Additionally, the lack of luteinizing hormone (LH) receptors in granulosa cells of the newly emerged follicles during the first day of wave emergence, the period to follicular deviation, thereby reducing follicular responsiveness to the induced LH surge (1). In heifers with three-wave cycles, around 57% of the estrous cycle occurs during stages that are unresponsive to the first GnRH administration. Consequently, failure to induce ovulation during these stages may allow premature regression of the corpus luteum (CL) before PGF_2α_ administration and result in early estrous expression (15, 16). Even when OVS-treated heifers respond to the first GnRH administration, rapid follicular turnover may cause the new DF to undergo atresia before PGF_2α_ administration, resulting in a growing follicle rather than a mature DF at the second GnRH administration and thereby reducing the probability of synchronized ovulation and conception (13). These findings emphasize that cycle stage, follicular maturation and turnover, LH receptor expression, and wave pattern are essential determinants of protocol success in heifers. Therefore, several strategies have been evaluated to promote the ovarian response to the first GnRH administration and to overcome stage-dependent variability in physiological responses at the beginning of OVS in heifers (17).

Among these strategies, presynchronization programs aim to control the physiological state prior to initiating TAI (18, 19). Additionally, adjusting the GnRH dose or analog type can improve the ovulatory efficiency. Ovulation in response to the first GnRH has consistently been associated with higher progesterone (P_4_) concentrations at the time of PGF_2α_ administration and improved pregnancy rate (P/AI), particularly in heifers that initiate the protocol without a functional CL or with low circulating P_4_ (20, 21). Several studies in dairy cattle and heifers have shown that increasing the dose of the GnRH analog (gonadorelin acetate) from 100 to 200 μg at the first hormonal treatment can enhance the ovulatory response to that injection by increasing the amplitude of LH release and subsequent follicular ovulation (22, 23). These findings highlight the potential of dose adjustment to address physiological variability at protocol initiation (22). Moreover, differences among GnRH analog types affect LH responsiveness and ovulation rate (4). Buserelin and lecirelin have been reported to be superior to gonadorelin, causing stronger responses (24–26).

However, the traditional administration of protein and peptide hormones faces several challenges, including variable pharmacokinetics, short half-life, rapid degradation and clearance, frequent injections, ineffective sustained release, and the need for relatively high doses to achieve consistent biological responses (27). These limitations may reduce the efficiency of treatment in livestock reproduction programs. Therefore, recent advances such as nanotechnology-based drug delivery systems have introduced promising alternatives to conventional hormonal formulations. Nanofabricated carriers can improve hormone bioavailability and stability, protect against enzymatic degradation, prolong systemic circulation, and allow controlled or sustained release of hormonal therapeutics (28). Their efficacy is attributed to unique properties, including small size and modifiable surface characteristics, that enhance pharmacokinetics and facilitate targeted delivery (29, 30).

Chitosan nanoparticles (CH-NPs) have been widely applied as promising carriers for several bioactive components. Their advantages include protecting these components from enzymatic degradation, increasing their bioavailability, providing sustained release, facilitating their transport across biological barriers, and improving cellular uptake (31, 32). In reproductive practices in livestock, chitosan (CH)-based nanocarriers, such as CH–dextran sulfate nanoparticles (CH–DS NPs) (33, 34), CH–alginate nanoparticles (CH–Alg NPs) (35), CH–sodium tripolyphosphate NPs (CH–TPP NPs) (36–40), and raw CH (41) have been utilized for GnRH delivery. These formulations may improve pharmacokinetic profiles and biological activity at reduced doses. Previous studies in rabbit does (36), goat does (39, 40), ewes (42), buffalo cows (37, 41), and dairy cows (34) have reported comparable or improved reproductive outcomes with a 50% reduction in nano-GnRH doses compared to conventional formulations. However, data regarding their potential effects in heifers are limited. Notably, administering the full conventional dose of nano-GnRH did not enhance ovarian characteristics such as follicular development and luteal characteristics compared to the half dose (39). Although the mechanism is still unclear, it is suggested that the prolonged release properties of nanocarriers may change hormone-receptor interactions at higher doses, indicating that dose reduction may be feasible when using nanocarriers for hormone delivery (39). Therefore, we selected a 50% reduced dose of the nanofabricated formulation of GnRH analogue (buserelin acetate; GnRH-BA) in the present study to evaluate whether it can maintain ovarian and reproductive responses in heifers under practical field conditions.

In this context, we hypothesized that integrating a reduced dose of nano–GnRH-BA into the OVS protocol may enhance the OVS outcomes in heifers by providing sustained stimulation of the hypothalamic-pituitary-ovarian (HPO) axis, reducing response variability, and improving synchronization efficiency and first conception rate (FCR). Therefore, the present study evaluates the impact of substituting conventional GnRH-BA with a nano–GnRH-BA formulation at 50% of the standard dose within the OVS protocol on ovarian activity and reproductive performance in Holstein heifers. This approach may help refine strategies for ovulation and estrous synchronization while promoting efficient and responsible hormone use in dairy production systems.

## 2 Materials and Methods

### 2.1 Preparation and characterization of nanofabricated formulation of GnRH-BA

#### 2.1.1 Fabrication of nanocarriers and nano-GnRH-BA

The fabricated nano–GnRH-BA was prepared by the ionic gelation method (36), based on ionic crosslinking between the cationic amino groups of chitosan (CH) and the anionic phosphate groups of TPP (tripolyphosphate), yielding CH–TPP nanocarriers. Pure CH (Sigma-Aldrich, Germany; degree of deacetylation 75%−85%; molecular weight 50,000–190,000 Da, viscosity-based) was dissolved in 1% (v/v) acetic acid to obtain a CH solution (0.1% w/v) under continuous magnetic stirring (800 rpm). A freshly prepared TPP solution (0.1% w/v; Sigma-Aldrich, Germany) was added dropwise to the CH solution at a TPP: CH ratio of 1:2 to promote spontaneous formation of CH–TPP NPs. A GnRH analogue solution (Receptal^®^, containing buserelin acetate, MSD, Intervet International GmbH, Unterschleißheim, Germany) was then introduced dropwise into the suspension while stirring at 800 rpm for 1 h. The pH was adjusted to 6.5 using 0.1 M NaOH. The resulting fabricated nano–GnRH-BA was aliquoted into sterile ampoules and stored at 4 °C until use (within 48 h).

#### 2.1.2 Characterization of nanocarriers and nano–GnRH-BA

For comparison, a Placebo formulation (blank nanocarrier) was prepared using the same nanofabrication procedure; in this case, Receptal^®^ was replaced by Dulbecco's phosphate-buffered saline (DPBS; Sigma-Aldrich, Germany). Hydrodynamic particle size (*D*_mean_) and Polydispersity Index (PdI) were determined for the fabricated nano–GnRH-BA and Placebo formulations using dynamic light scattering (DLS) with a Zetasizer Nano ZS (Malvern Instruments Ltd., Worcestershire, UK). Measurements were performed at room temperature, and each sample was analyzed in triplicate (*n* = 3); results are reported as mean ± standard deviation (SD).

Surface morphology of nano–GnRH-BA and placebo was examined by scanning electron microscopy (SEM; TESCAN MIRA3, Brno, Czech Republic). Samples were drop-casted onto a microelectronic-grade silicon (Si) wafer and allowed to dry before imaging. Micrographs were acquired at an accelerating voltage of 1.0 kV using an In-Beam SE detector (TESCAN, Brno, Czech Republic). Images were recorded at multiple randomly selected positions across the sample area, and representative micrographs were selected to illustrate typical particle morphology and size features.

#### 2.1.3 Evaluation of drug loading efficiency

Drug loading efficiency (DLE%) of nano–GnRH-BA was determined by quantifying the free (non-associated) drug in the supernatant after centrifugation using HPLC. Briefly, 1.0 ml of nano–GnRH-BA was transferred into a microcentrifuge tube and centrifuged at 4 °C for 20 min at 4,000 rpm (≈1,500 × g) using an Eppendorf 5,424 R refrigerated microcentrifuge equipped with an FA-45-24-11 fixed-angle rotor (Eppendorf, Hamburg, Germany). After centrifugation, 200 μl of the supernatant was collected and diluted two-fold with a diluent consisting of a 1:1 (v/v) mixture of mobile phases A and B. In parallel, an uncentrifuged aliquot of the same nano–GnRH-BA formulation was processed identically (200 μl sample diluted two-fold with the same diluent) and analyzed as the total drug content (100% reference). The total drug content measurement served as a quality control check against the nominal (target) drug concentration. Measurements were performed in triplicate using independent sample preparations (*n* = 3), and the results were reported as mean ± SD.

Chromatographic analysis was performed on an Agilent 1,260 HPLC system (Agilent Technologies, Waldbronn, Germany) equipped with a DAD-UV detector. Separation was achieved on a YMC MeteoricCore C18 column (150 mm × 4.6 mm, 2.7 μm; YMC Co., Ltd.) maintained at 40 °C. UV detection was performed at 280 nm, and the injection volume was 10 μl. The mobile phase consisted of (A) 0.1% (v/v) trifluoroacetic acid (TFA) in water and (B) acetonitrile. Elution was performed using the following gradient program (expressed as %A): 0.0 min, 90% A; 6.0 min, 50% A; 7.0 min, 5% A; 7.5 min, 5% A; 8.0 min, 90% A; 10.0 min, 90% A. Drug concentrations were calculated using an external calibration curve prepared in the same diluent.

### 2.2 Animals and management practices

The current study was conducted across two farms. The first farm is in Tisztaberek (≈ approximately 47.95° N, 22.8° E) in Szabolcs-Szatmár-Bereg County, eastern Hungary, while the second is situated in Bugyi (≈ 47.13° N, 19.18° E) in Pest County, Central Hungary. This study involved 210 Holstein heifers with an average age of 13.8 ± 1.5 months. All eligible heifers available during the study period were enrolled. From the first farm, 120 heifers were enrolled, with 68 assigned to the treatment group and 52 to the control group. The second farm provided 90 heifers, including 60 in the treatment group and 30 heifers in the control group. Heifers were housed in open free-stall barns equipped with cubicles. Additionally, barns were equipped with headlocks to facilitate handling and experimental manipulation.

All heifers were fed a nutritionally balanced total mixed ration (TMR), specifically formulated according to their maintenance and growth requirements. Before enrolling in the study, each animal underwent an examination to confirm its health status and to ensure the absence of reproductive disorders. To facilitate management and monitoring, animals were enrolled in experiments in groups of 30–40 heifers, with enrollments occurring every 2 weeks. The study was conducted during the winter season (from December to March) to mitigate climate variation and prevent heat stress. 30 heifers did not undergo ultrasound examination on Day 9 due to farm management constraints, although they received the full treatment protocol. Therefore, these heifers were excluded from the ultrasonographic analysis. However, they were included in the fertility outcome analyses (FCR and PL) as an indicator of treatment response.

Heifers from both farms were managed under identical conditions and followed the same treatment protocol and handling procedures to minimize the potential farm-related effect.

### 2.3 Experimental design

A total of 210 healthy Holstein heifers were subjected to ovulation synchronization using the OVS protocol. The procedure commenced with an initial injection of GnRH (Receptal^®^, day 0), followed by administration of PGF_2α_ (500 μg/head of Cloprostenol, Veyx Pharma, Germany) on day 7. A second injection of GnRH was administered 48 h after PGF_2α_ to induce ovulation.

**Figure 1:**
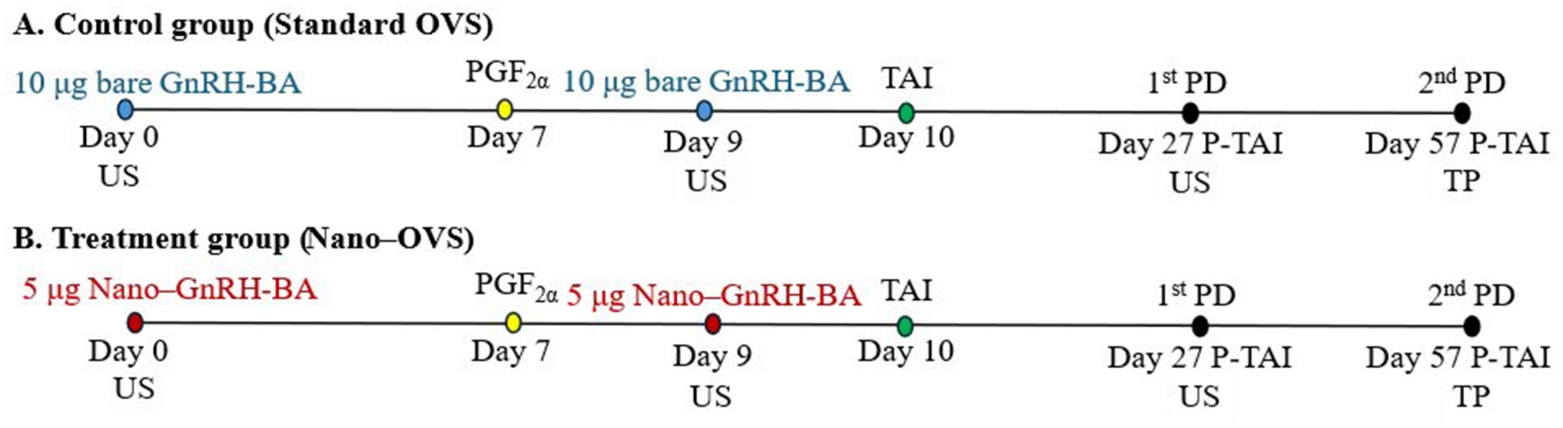
Experimental design of Ovsynch protocols in Holstein heifers. The control group received the standard OVS protocol (*n* = 82), which involved using the typical dose of bare GnRH-BA (10 μg/ administration). The treatment group was subjected to the Nano–OVS (*n* = 128), which utilized a half-dose of nano–GnRH-BA (5 μg/ administration). OVS, standard ovsynch protocol; GnRH-BA, gonadotropin releasing hormone analogue (buserelin acetate); Nano–GnRH-BA, nanofabricated GnRH-BA with CH–TPP nanocarrier; PGF_2α_, prostaglandin; TAI, fixed-timed artificial insemination; TP, transrectal palpation; P-TAI, post-TAI; PD, pregnancy diagnosis; US, ultrasonography.

The heifers were assigned to two groups, as shown in Figure 1, each receiving a different GnRH dose and formulation within the OVS protocol. The first group (control group, *n* = 82) received the standard OVS protocol, which included the recommended dose and formulation of GnRH (10 μg GnRH, Receptal) on days 0 and 9. The second group (treatment group, *n* = 128) received the same OVS protocol with a reduced dose (50% of the recommended dose) as nano–GnRH-BA (5 μg GnRH, nanofabricated GnRH) on days 0 and 9; all injections were administered intramuscularly. Unequal group sizes resulted from the availability under commercial farm conditions, not from a predetermined allocation ratio. 30 heifers did not undergo ultrasound examination on Day 9 due to farm management constraints, although they received the full treatment protocol. Therefore, these heifers were excluded from the ultrasonographic analysis. However, they were included in the fertility outcome analyses (FCR and PL) as an indicator of treatment response. Artificial insemination (AI) was performed 16–20 h following the second GnRH injection, using high-quality frozen-thawed semen.

### 2.4 Ultrasound examination

The assessment of the ovarian activity and pregnancy diagnosis (PD) was performed using transrectal ultrasonography with an ultrasound device equipped with a linear transducer (Easi-Scan IV, IMV, France). The ovarian activity was evaluated three times: initially on day 0 as a baseline before the first GnRH injection, on day 9 before the second injection of GnRH to assess the response of PGF_2α_ injection administered on day 7, and to examine the follicular dynamics before the second GnRH and also between days 27 and 30 post-TAI to evaluate the luteal activity in response to the second GnRH injection. The evaluation of ovarian activity involved determining the total number of follicles and measuring their diameters, classifying follicles into subordinate (>3 mm to < 10 mm) and large (>10 mm), and assessing the number of CL and their largest diameters as indicators of luteal activity. Additionally, on days 0 and 9, the uterus was examined to detect uterine fluid, assess uterine tone, and evaluate the estrus induction rate (EIR). This rate was defined by the proportion of heifers exhibiting intrauterine fluid and uterine tone indicative of estrus on day 9, relative to the total number of examined heifers.

Moreover, PD was conducted between days 27 and 30 post-TAI. This diagnosis was based on four specific criteria: (1) the presence of an embryo, (2) the recognition of the embryo's heartbeat, (3) the observation of amniotic and allantoic fluid, and (4) the detection of a CL on the ovary. A pregnancy was considered positive only if all criteria were met simultaneously. To assess pregnancy loss (PL), a subsequent examination was performed by farm technicians between days 57 and 60 post-AI using transrectal palpation to confirm pregnancy by noting a symmetrically enlarged uterine horn and fluid fluctuation within it.

The FCR was calculated using the formula FCR = [number of heifers conceived on day 27–30/ total number of inseminated heifers × 100], while PL was determined using PL = [number of heifers that lost pregnancy after day 30/ number of heifers that were pregnant × 100].

### 2.5 Statistical analysis

All analyses were performed using IBM SPSS Statistics (Version 24, IBM, SPSS Statistics Inc., NY, USA). Charts and heatmaps were generated using GraphPad Prism (Version 9, GraphPad Software, San Diego, CA, USA). Before modeling, the normality of continuous variables was assessed using Q-Q plots of the residuals. When necessary, appropriate data transformations were applied to meet the model's assumptions of normality and homoscedasticity.

Continuous variables, including follicle and CL diameters, were analyzed using Linear Mixed Models (LMM) via the MIXED procedure. Treatment (OVS and nano–OVS), time (day 0, 9, and 27 post-AI), and their interaction were included as fixed factors. Baseline measurements at day 0 were included as covariates to adjust for initial differences in ovarian activity among heifers. The farm was incorporated as a random intercept to account for potential environmental and management-related variation. Repeated measurements across days were modeled within heifer ID using a first-order autoregressive [AR(1)] covariance structure to account for within-animal correlation over time. Estimated marginal means (EMmeans) were evaluated for all fixed factors, and pairwise comparisons were performed using a Bonferroni adjustment to control for multiple testing.

Count data, such as the numbers of follicles and CL, were analyzed using generalized linear mixed models (GLMMs) with a Poisson distribution and a log link function. Fixed effects included treatment, time, their interaction, and the corresponding baseline values at day 0, while random intercepts included farm and heifer ID nested within farm. Repeated measures across days were also specified.

Results are presented as original (back-transformed) EMmeans ± standard error (SE) to present the original count scale for facilitating biological interpretation. Bonferroni-adjusted pairwise comparisons were conducted after identifying significant fixed effects.

Binary outcomes, including EIR, FCR, and PL, were analyzed using GLMMs with a binomial distribution and a logit link function. Treatment was included as a fixed effect, with farm effect incorporated to adjust for clustering at the farm level. Results are reported as odds ratios (OR) with 95% confidence intervals (CIs) to provide interpretable estimates of relative risk between treatment groups.

Additionally, the association between ovarian activity across experimental days and subsequent fertility outcomes was assessed using Spearman's rank correlation. Correlations were calculated separately for each treatment group using the split file function. Differences were considered statistically significant at *P* ≤ 0.05, and 0.05 < *P* ≤ 0.10 was interpreted as a tendency.

## 3 Results

### 3.1 Nanocarriers and nano–GnRH-BA characteristics

Data summarized in Table 1 show that the *D*_mean_ was 293.3 ± 4.1 nm for placebo and 292.9 ± 5.9 nm for nano–GnRH-BA, indicating nearly identical particle sizes between the two formulations. Both samples showed moderate polydispersity, with PDI values of 0.41 ± 0.04 for the placebo and 0.33 ± 0.08 for nano–GnRH-BA. Additionally, the DLE% for nano–GnRH-BA was 90.7 ± 0.5%.

**Table 1:** Physicochemical characteristics, including hydrodynamic particle size (*D*_mean_), Polydispersity Index (PdI), and drug loading efficiency (DLE %) of placebo and nano–GnRH-BA.

Formulations	*D*_mean_ ±SD (nm)	PdI ±SD	DLE ±SD (%)
Placebo	293.3 ± 4.1	0.41 ± 0.04	ND
Nano–GnRH-BA	292.9 ± 5.9	0.33 ± 0.08	90.7 ± 0.5

ND, not detected; SD, standard deviation; Nano-GnRH, GnRH–loaded chitosan nanoparticles.

**Figure 2:**
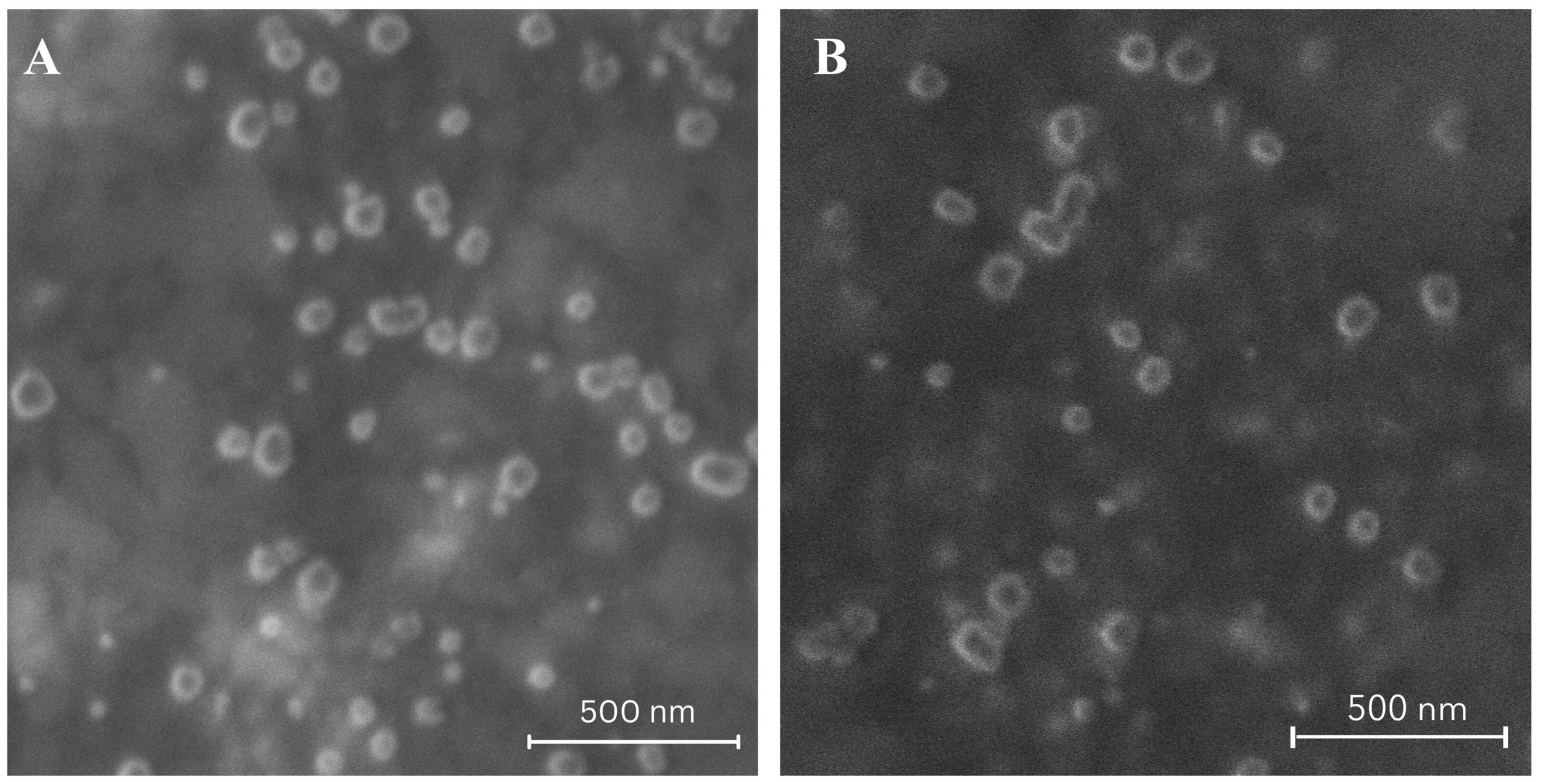
Scanning electron microscope (SEM) images of the placebo **(A)** and nano–GnRH-BA **(B)** formulations, with a 500 nm scale bar, were captured at 1.0 kV acceleration voltage using an In-Beam SE detector at 25 °C.

Representative SEM micrographs (Figure 2) depict that both formulations consisted of nanoscale particles with a predominantly near-spherical morphology. Based on the scale bar, the apparent particle dimensions were below 100 nm.

### 3.2 Ovarian activity

Table 2 presents baseline-adjusted estimates of the ovarian dynamics, including follicular and luteal characteristics, observed across three distinct time points between groups. At baseline (day 0), heifers treated with nano–OVS had a significantly higher total number of follicles compared with those in the standard OVS group (2.5 ± 0.1 vs. 2.2 ± 0.1, respectively; *P* = 0.05). No significant differences were observed in the number and diameter of subordinate follicles, large follicles, as well as CL (*P* > 0.05).

**Table 2:** The impact of treatment (nano–OVS protocol vs. standard OVS protocol as a control) on ovarian response, including follicular and luteal characteristics, in holstein heifers across different time points (day 0, day 9, and day 27 post-AI).

Variables	Experimental Groups		*P*-Value
	Control	Treatment	
Before the 1^st^ GnRH administration (day 0)			
Number of heifers	82	98	
Total number of follicles	2.2 ± 0.1^B^	2.5 ± 0.1^A^	0.05
Subordinate (≥3– < 10 mm) follicle number	1.7 ± 0.2	1.8 ± 0.2	0.42
Subordinate (≥3– < 10 mm) follicle diameter	6.1 ± 0.2	5.8 ± 0.1	0.37
Large (≥10 mm) follicle number	1.3 ± 0.05	1.3 ± 0.04	0.96
Large (≥10 mm) follicle diameter	14.0 ± 0.6	14.4 ± 0.6	0.42
CL number	0.8 ± 0.05	0.8 ± 0.05	0.88
CL diameter (mm)	16.7 ± 1.2	16.5 ± 1.2	0.92
Response to PGF_2α_ administration (day 9)			
Number of heifers	82	98	
Total number of follicles	1.7 ± 0.1	1.7 ± 0.1	0.46
Subordinate (≥3– < 10 mm) follicle number	1.4 ± 0.2	1.3 ± 0.1	0.32
Subordinate (≥3– < 10 mm) follicle diameter	4.7 ± 0.2^B^	6.6 ± 0.2^A^	< 0.001
Large (≥10 mm) follicle number	1.3 ± 0.05	1.2 ± 0.04	0.13
Large (≥10 mm) follicle diameter	16.3 ± 0.7^B^	17.5 ± 0.71^A^	0.05
Preovulatory follicle diameter (mm)	16.8 ± 1.3^B^	18.9 ± 1.4^A^	0.007
CL number	0.3 ± 0.05	0.4 ± 0.05	0.10
CL diameter (mm)	4.1 ± 1.2	5.5 ± 1.2	0.21
At pregnancy diagnosis (day 27 post-TAI)			
Number of heifers	82	98	–
Total number of follicles	ND	ND	–
CL number	0.9 ± 0.07	0.9 ± 0.06	0.97
CL diameter (mm)	18.3 ± 1.4^B^	21.3 ± 1.3^A^	0.04

GnRH, gonadotropin-releasing hormone; PGF_2α_, prostaglandin; CL, corpus luteum. Means with different uppercase letters (A, B) within the same row are significantly different.

On day 9, in response to PGF_2α_ administration and before the second GnRH, no significant differences were recorded between groups in total follicle count, subordinate follicle number, large follicle number (*P* > 0.05), or CL number and diameter (all *P* > 0.05). In contrast, the diameter of subordinate and large follicles was significantly larger in the treated heifers than in controls (6.6 ± 0.2 vs. 4.7 ± 0.2 mm, and 17.5 ± 0.7 vs. 16.3 ± 0.7, respectively; *P* < 0.05). Similarly, the preovulatory follicles reached a larger diameter in treated heifers (18.9 ± 1.4 mm) compared to control heifers (16.8 ± 1.3 mm; *P* = 0.007).

Only luteal characteristics were assessed on day 27 post-TAI. At this point, the nano–OVS group had a significantly larger CL diameter (21.3 ± 1.3 mm) compared with the control group (18.3 ± 1.4 mm; *P* = 0.04), while the number of CL remained comparable between groups (*P* > 0.05)

**Figure 3:**
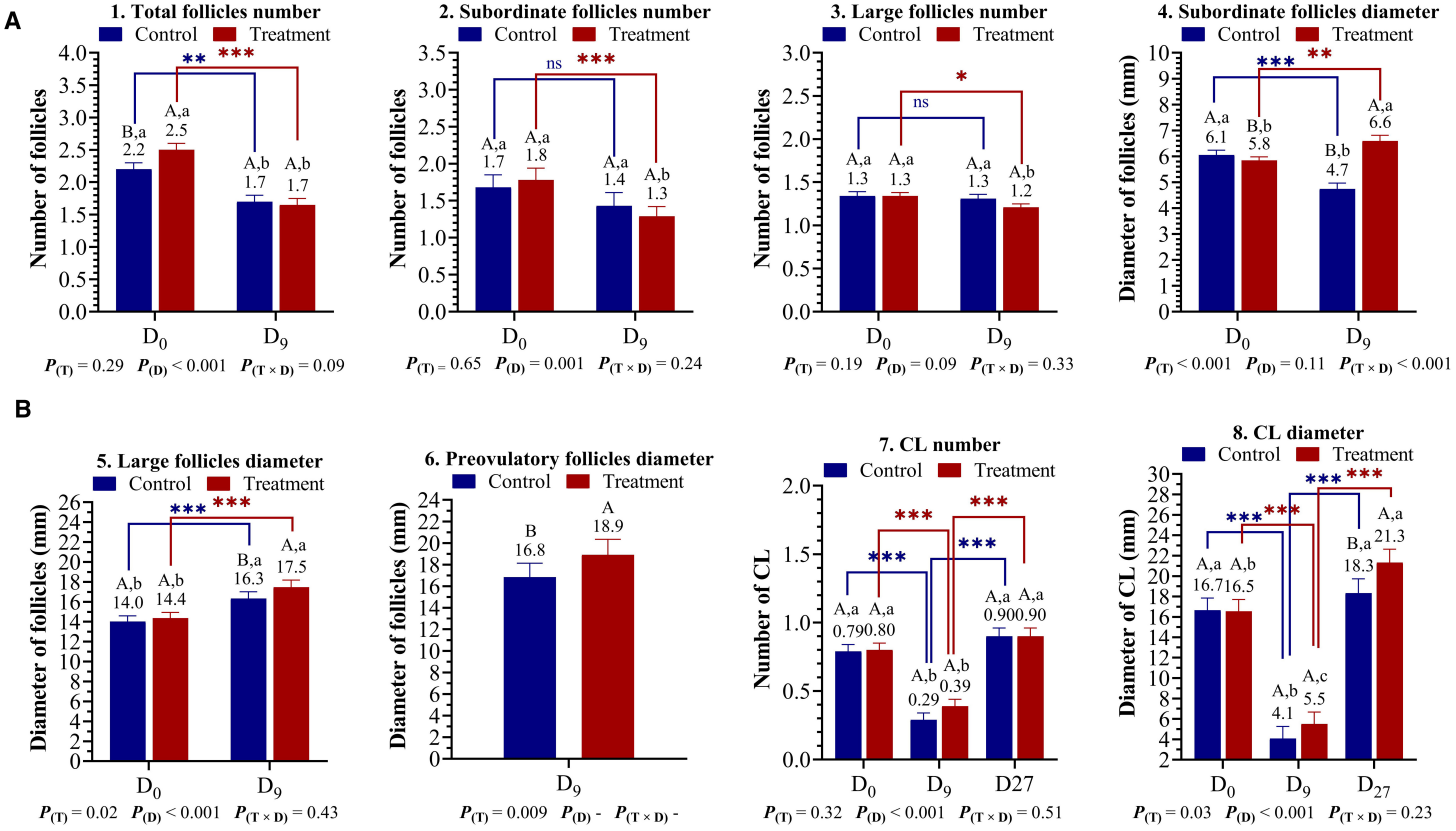
Impact of treatment (nano–OVS protocol vs. standard OVS protocol as a control) and time (days 0, 9, and 27 post-AI) on ovarian activity includes follicular dynamics such as the number of (1) total follicles, (2) subordinate follicles, and (3) large follicles. It also consists of the diameter of (4) subordinate follicles, (5) large follicles, and (6) preovulatory follicles. Additionally, luteal dynamics include (7) CL number and (8) CL diameter. Bars marked with different uppercase letters **(A, B)** indicate significant differences between treatments on the same day. In contrast, bars marked with varying letters of lowercase (a, b, c) indicate significant differences among days within the same treatment (*P* < 0.05). Asterisks indicate *P*-values of significant differences among days within the same treatment: *P < 0.05, and **P < 0.01.

Comparisons between experimental groups over time regarding follicular and luteal dynamics are depicted in Figure 3. Both groups showed a significant decline in total follicle count by day 9 of the protocol relative to day 0 (*P* < 0.001) (Figure 3.1). Subordinate follicle numbers (Figure 3.2) were similar at baseline; however, only the treatment group showed a significant decline by day 9 (*P* = 0.05), while the control group exhibited a non-significant numerical decrease (*P* > 0.05). A similar pattern was observed between days for large follicle numbers (Figure 3.3), with comparable baseline values, followed by a significant slight reduction in the treatment group (*P* < 0.05) and a non-significant decline in the control group (*P* > 0.05).

Regarding follicular diameter (Figure 3.4), by day 9, the diameter of subordinate follicles markedly decreased in the control group, while it significantly increased in the treatment group. For large follicles (Figure 3.5), both groups exhibited comparable diameters on day 0, both measuring about 14 mm. By day 9, follicular diameters increased in both groups, with the treatment group reaching a significantly higher measurement of 17.5 mm compared with the control group (16.3 mm; *P* < 0.001). Preovulatory follicles on day 9 were approximately 2 mm larger in the treatment group than in the control group (*P* < 0.05) (Figure 3.6).

In terms of luteal dynamics (Figures 3.7, 3.8), both groups showed a decrease in the numbers and diameters of the CL on day 9, with no significant differences between groups (*P* > 0.05). By day 27 post-AI, both groups showed a significant increase in the average number of CL (both 0.9) compared with day 9, without a significant difference between groups (*P* > 0.05). However, the treatment group demonstrated a significantly larger CL diameter (*P* < 0.05), approximately 3 mm greater than that of the control group (21.3 vs. 18.3, respectively).

### 3.3 Reproductive performance outcomes

The reproductive performance outcomes were consistent with the ovarian follicular dynamics and luteal characteristics observed in this study (Table 3). The treatment group exhibited a significantly higher EIR compared with the control group (OR = 1.72; *P* = 0.03). Treated heifers also showed a higher FCR (OR = 1.33) than controls; however, the difference did not reach statistical significance (*P* = 0.32). Notably, PL was significantly reduced in treated heifers (*P* = 0.01; OR = 0.15) compared with their control counterparts.

**Table 3:** The effects of treatment (nano–OVS protocol vs. standard OVS protocol as a control) on reproductive performance outcomes include the estrus induction rate (EIR), the first conception rate (FCR), and the pregnancy loss rate (PL).

Parameters	*N* (%)	B ±SE	OR	95% CIs	*P-*value
EIR					
Control	54/82 (65.9%)	Ref.	–	–	–
Treatment	77/98 (78.6%)	0.54 ± 0.2	1.72	1.07–2.75	0.03
FCR					
Control	33/82 (40.2%)	Ref.	–	–	–
Treatment	61/128 (47.7%)	0.28 ± 0.3	1.33	0.76–2.33	0.32
PL					
Control	7/33 (21.2%)	Ref.	–	–	–
Treatment	8/61 (13.1%)	−1.92 ± 0.8	0.15	0.03–0.68	0.01

B, regression coefficient; SE, standard error; 95% CIs, 95% confidence intervals.

### 3.4 Correlation between ovarian dynamics and fertility outcomes

**Figure 4:**
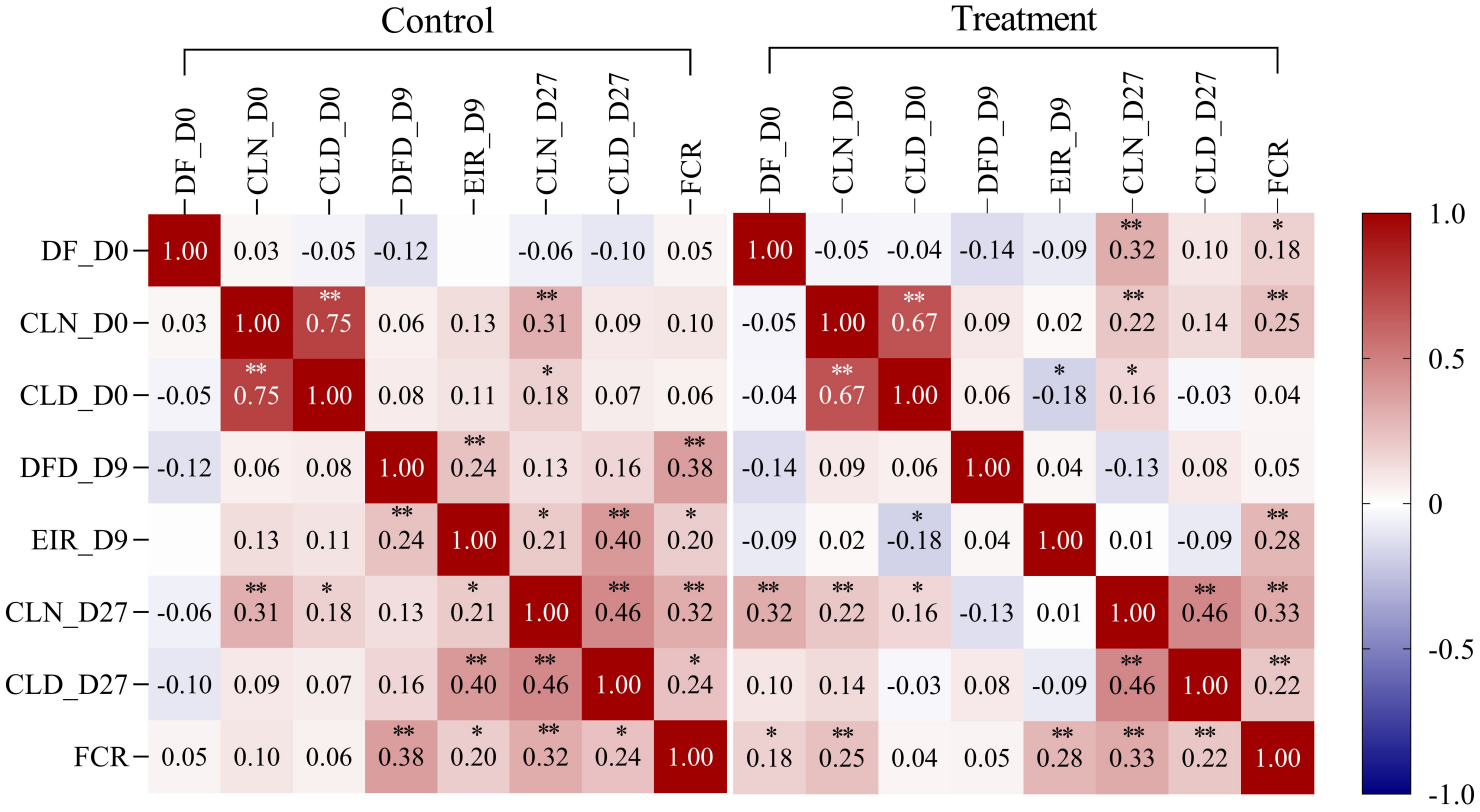
Comparative Spearman correlation heatmap of the ovarian activity, including follicular dynamics and luteal dynamics, and fertility outcomes across experimental duration for control and treatment groups. Sperman's correlation coefficients are exhibited presence of dominant follicle on days 0 (DF_D0), corpus luteum number on days 0 and 9 (CLN_D0, CLN_D9), corpus luteum diameter on days 0 and 9 (CLD_D0, CLD_D9), dominant follicle diameter on day 9 (DFD_D9) estrus induction on day 9 (EIR_D9), and the first conception rate (FCR). The scale ranges from−1 (dark blue) to +1 (red). Asterisks indicate significant differences: *P < 0.05, and **P < 0.01.

The association between ovarian activity and fertility outcomes in both treatment and control groups is depicted in Figure 4. In the control group, baseline ovarian structure measured on day 0 of the standard OVS protocol was not significantly associated with FCR. Neither the presence of DF (*r* = 0.05), the presence of CL (*r* = 0.10), nor CL diameter (*r* = 0.06) exhibited significant correlation with FCR (*P* > 0.05). Conversely, FCR was mainly associated with ovarian traits observed later in the protocol. For instance, the DF diameter on day 9 showed a modest positive correlation with FCR (*r* = 0.38; *P* < 0.01). Estrous expression or EIR on the same day also exhibited a positive association with FCR (*r* = 0.20; *P* < 0.05) and subsequent CL number and diameter (*r* = 0.21; *P* < 0.05 and *r* = 0.40; *P* < 0.01, respectively). Additionally, luteal characteristics measured at PD were moderately associated with FCR, including CL number and CL diameter on day 27 post-AI (*r* = 0.32; *P* < 0.01 and *r* = 0.24; *P* < 0.05, respectively).

In the heifers subjected to nano–OVS, slightly different correlation patterns emerged. Baseline ovarian characteristics exhibited significant correlations with FCR, whereas in the control group, they did not. Both DF (*r* = 0.18; *P* < 0.05) and CL (*r* = 0.25; *P* < 0.01) on day 0 were positively associated with FCR. Estrous expression on day 9 remained an essential predictor for fertility (*r* = 0.28; *P* < 0.01) but showed a weaker relationship with subsequent CL characteristics (*P* > 0.05). Also, luteal characteristics at PD, such as the number of CL (*r* = 0.33; *P* < 0.01) and CL diameter (*r* = 0.22; *P* < 0.01), were positively associated with FCR. Notably, the association between DF diameter on day 9 and FCR was weaker in the treatment group (*r* = 0.05; *P* > 0.05) than in the control group.

## 4 Discussion

The current study was designed to evaluate the impact of administering a 50% reduced dose of nano–GnRH-BA within the OVS protocol on estrous synchronization and reproductive performance in Holstein dairy heifers. Ovarian activity, fertility outcomes, and their potential association were assessed.

In this study, the *D*_mean_ values for the placebo and nano–GnRH-BA formulations were ~293.3 nm and ~292.9 nm, respectively. Interestingly, the nano–GnRH-BA formulation showed almost unchanged *D*_mean_ compared to placebo (unconjugated CH-NPs). This observation contrasts with previous studies that reported an increase in particle size after the loading of GnRH into CH-NPs compared to unconjugated CH-NPs (36–38, 43, 44). Only one study reported a significant reduction of particle size from ~126 nm for GnRH-loaded CH-NPs to ~94 nm for unloaded CH-NPs (40). The relatively unchanged size obtained following GnRH loading in the present study may be attributed to enhanced electrostatic interactions that increase crosslinking density, promoting the formation of a highly compact matrix and enhancing the structural stability of the CH-NPs system (45).

CH-NPs have been widely utilized as promising carriers for several bioactive components due to their functional properties (31, 32). These properties are influenced by the physical characteristics of NPs, particularly particle size, which is a crucial determinant of penetration of biological barriers and intracellular uptake (31). For instance, smaller NPs (~100 nm) have been shown to exhibit greater arterial uptake than larger NPs (>200 nm) (46). Conversely, other studies have suggested that NPs within the 200–350 nm range improve uptake by mucosal epithelial cells (47). Based on these findings, the nano–GnRH-BA formulation with a size of ~293 nm remains suitable for intramuscular administration in heifers.

The PdI also tended to decrease from 0.42 in the placebo to 0.33 in the nano–GnRH-BA formulation, indicating increased NPs homogeneity and a more uniform size distribution after GnRH conjugation. Lower PdI values, ranging from 0 to 0.5, are widely considered indicators of a homogeneous suspension and greater colloidal stability. In contrast, PdI values exceeding 0.5 may suggest particle aggregation or heterogeneous size distribution (48, 49). In general, PdI values lower than 0.4 are considered acceptable for NPs-based drug delivery systems, as they are associated with better physical stability and a reduced risk of aggregation (50).

These observations were further confirmed by SEM imaging, which showed well-distributed, non-aggregated spherical particles. Image acquisition for nano–GnRH-BA was more challenging, showing lower contrast than for the placebo, which may be related to the presence of benzyl alcohol as an excipient in the Receptal formulation. During imaging, the samples could not be brought into sharp focus. The NPs appeared to be embedded in or partially covered by an amorphous, polymer-like layer, attributed to excess CH that did not assemble into discrete NPs. With continued electron-beam exposure, this surrounding material showed beam-induced degradation, resulting in smearing or softening of features and further loss of image sharpness. As a result, sharper images could not be obtained under the applied conditions. Despite the lower contrast observed for nano–GnRH-BA, both formulations showed a similar overall appearance. However, a quantitative comparison between the two formulations was not feasible under the SEM conditions applied.

At higher acceleration voltages, contrast decreases due to the higher penetration depth of the incoming electrons, and degradation occurs more rapidly. In contrast, lower acceleration voltages reduce beam focus, which also leads to a loss of resolution, although beam-induced damage may be less pronounced.

Notably, smaller particles were observed by SEM compared to DLS measurements, which may be attributed to the *D*_mean_ measured in DLS, including the hydration layer. The relatively high PdI (~0.4) may therefore reflect the presence of unbounded CH chains in the surrounding media. Additionally, the use of CH with low molecular weight could increase the number of free unbounded polymer chains at a given concentration, potentially contributing to a broader size distribution. Comparison of the two techniques shows sub-100 nm particle features by SEM, while DLS yielded mean hydrodynamic diameters of ~293 nm for both formulations. This reflects the different measurement states: SEM visualizes the dried material on the substrate, whereas DLS reports the hydrodynamic diameter in dispersion, including the solvated particle core, any hydration layer, and the polymer corona. In CH–TPP systems, part of the CH can remain as non-particulate chains (or loosely associated/coacervated polymer) that do not form discrete NPs but stay associated with the particle surface and extend into the aqueous phase. These solvated chains can increase the apparent DLS size and broaden the distribution, while supporting colloidal stabilization through steric and electrosteric contributions. Taken together, the DLS and SEM data indicate comparable particle attributes for Placebo and nano–GnRH-BA; the incorporated GnRH did not change particle size distribution or qualitative morphology under the applied conditions.

Moreover, these results are consistent with the high DLE% for GnRH (~90.7%) observed in our study, indicating effective incorporation of the peptide into the CH matrix without compromising the nanocarrier's structural stability. DLE% values below 100% are expected and may result from the finite binding capacity of the CH matrix and an adsorption/partition equilibrium, leaving a small free fraction of the peptide in the surrounding continuous phase. This high DLE% may be attributed to the formation of a denser composite matrix, which enhances the structural integrity and increases hormone entrapment capacity through stronger electrostatic interactions between the peptide and the CH-NPs (50). Such a high DLE suggests efficient utilization of the loaded hormone, which is an important factor in enhancing delivery performance. Moreover, the strong interaction between the hormone and nanocarriers may contribute to controlled release, increased bioavailability, enhanced targeting efficiency, and a potential reduction in the required dose (51). Our results are consistent with previous reports that have demonstrated high DLE values for various GnRH analogs loaded into the CH-NPs, typically ranging from 87.7 to 91.2% (36–38, 40, 42–44).

Taken together, the observed near-spherical morphology, particle size, acceptable PdI, and high DLE indicate that the CH-NPs maintained the structural stability after GnRH conjugation. These characteristics collectively support the suitability of nano–GnRH-BA formulation for potential application within reproductive synchronization protocols.

In the current study, incorporating nano–GnRH-BA within the OVS protocol significantly influenced ovarian dynamics and reproductive performance in dairy heifers. All measurements were adjusted for baseline values at day 0 to control the potential differences in ovarian structure among heifers, ensuring that the observed effects were more likely attributable to the treatment effect than to pre-existing variations in ovarian status. Following protocol initiation, particularly on day 9 after PGF_2α_ administration, heifers synchronized with nano–OVS had larger subordinate, large, and preovulatory follicle diameters compared with those receiving standard OVS. These observations indicate that follicular recruitment and growth progressed more effectively during synchronization in the nano–OVS group.

The superiority of nano–OVS may be associated with the proposed pharmacokinetics benefits of nanofabricated GnRH with CH-NPs (31). However, hormone release kinetics and direct endocrine responses were not evaluated in our study. Previous reports have suggested that the encapsulation process may protect GnRH from rapid enzymatic degradation and enable sustained hormone release, thereby maintaining stimulation of the HPO axis and extending LH secretion (31, 36). This prolonged LH availability promotes final follicular maturation and the development of the competent ovulatory follicle (52, 53). Follicle size at AI is an essential factor influencing estradiol (E_2_) concentrations, ovulation quality, and the subsequent endocrine environment that supports fertilization and the establishment of pregnancy (54). Larger preovulatory follicles typically produce higher E_2_ levels, thereby enhancing estrous expression, LH surge responsiveness, and oocyte competence, ultimately leading to improved fertility outcomes (54). Consequently, heifers with adequately developed ovulatory follicles tend to achieve higher fertility outcomes in TAI synchronization protocols.

This mechanism may also explain the observed correlation patterns between DF diameter on day 9 and FCR in both groups. In the standard OVS group, DF diameter on day 9 was significantly correlated with FCR, whereas no correlation was observed in the nano–OVS group. Larger DFs are associated with a larger CL and increased P_4_ production, which, in turn, are related to improved fertility outcomes and reduced PL (54, 55). This inverse correlation may reflect variability in follicular dynamics between groups. A possible explanation is that nano–OVS promoted more uniform follicular response; however, the mechanisms underlying this finding were not directly evaluated.

These observations align with previous studies in other livestock species. GnRH-loaded CH-NPs have been shown to induce ovulation effectively at reduced doses in rabbit does, with earlier LH surges compared with conventional GnRH administration (36). Similarly, in anestrous buffalo cows and heat-stressed dairy cows, incorporating GnRH-CH-NPs into the OVS protocol significantly enhanced follicular development, increased DF size and the number of ovarian follicles, and improved fertility outcomes (37, 41). In goats, nanofabricated GnRH increased follicle numbers and diameter, along with E_2_ and nitric oxide (NO) levels, which are associated with improved ovarian blood flow and steroidogenesis due to their vasodilatation effects (39, 56). Collectively, these studies support the hypothesis that nano–GnRH-BA may enhance ovarian responsiveness, although the hormone stability and bioavailability were not measured in the current study.

Luteal dynamics further supported the beneficial effects of nano–OVS. Although both groups exhibited effective luteolysis following PGF_2α_ administration, this indicates effective luteolysis on day 7, as evidenced by regression of luteal tissue (57). However, a numerically larger CL diameter was observed in the nano–OVS group (5.2 mm) compared with the standard OVS group (4.0 mm; *P* > 0.05) during the follicular phase. Because P_4_ was not included in the present study, the physiological causes of this difference remain speculative. Previous reports have demonstrated that the increased P_4_ concentrations during the follicular phase are known to enhance follicular turnover and support the emergence of a new follicular wave (58, 59). These impacts may also be linked to previously reported mechanisms, including associations with increased DF diameter (33, 37) and elevated E_2_, NO concentrations, and ovarian blood flow (39). However, these variables were not assessed in the current study.

These improvements were also reflected in EIR, which was significantly higher in the nano–OVS group (79%) compared with the standard OVS group (66%; *P* = 0.03). Comparable findings have been observed in anestrous buffalo, where GnRH-CH-NPs significantly increased EIR compared with conventional GnRH (80 vs. 50%, respectively) (37). Additionally, Amin et al. (41) reported a significant increase in EIR (87%) in acyclic buffalo cows treated with GnRH mixed with CH within the OVS protocol compared with conventional OVS (27%). These positive effects have previously been attributed to the enhanced stability, bioavailability, and biological activity of GnRH when conjugated to CH and CH-NPs (29, 41).

Interestingly, both groups showed a positive correlation between EIR and FCR, with a slightly higher correlation in the nano–OVS group. This difference may be associated with an increased DF diameter, which could elevate circulating E_2_ levels and thereby enhance estrous expression. As reported by Souza et al. (60), supplementation with E_2_ within the OVS protocol at the second GnRH administration increased estrous expression. It resulted in greater fertility compared to cows that did not show estrus. It has been reported that the likelihood of pregnancy is associated with both the occurrence and intensity of estrus around the time of AI in dairy cattle (61–63). This may be explained by the fact that, compared with cows that do not exhibit estrus yet still experience normal ovulation and luteal development, the incidence of estrus around AI can alter gene expression related to endometrial receptivity and embryonic development. These alterations are related to genes involved in the preimplantation phase of conceptus development (64).

Following TAI and during early gestation (>27 days post-AI), heifers subjected to nano–OVS had larger CL than those in the standard OVS group. The improved luteal development observed with the application of nano–OVS may be explained by enhanced luteinization of granulosa and theca cells after ovulation (54). GnRH administration around ovulation time may enhance CL function by triggering LH release and promoting subsequent luteinization of follicular cells (29).

Similar findings have been reported in dairy cattle under heat stress, where nano–GnRH improved P_4_ levels despite reduced dosing (38). In acyclic buffalo, GnRH combined with CH within an OVS protocol increased luteal function by doubling P_4_ levels (2.3 ng/ml vs. 1.2 ng/ml) and the conception rate (77 vs. 50%) compared with standard OVS (41). Likewise, GnRH-loaded CH-NPs increased CL diameter by 6 mm (21 vs. 15 mm, respectively) and enhanced P_4_ levels, which were associated with improved CR (75 vs. 40%, respectively) compared with bare GnRH in buffalo cows during the low breeding season (37). Similar impacts have also been observed in goats, where nano–OVS markedly increased CL diameter (8.0 mm) compared to conventional OVS (7.6 mm), along with increased luteal blood flow and luteal function, as evidenced by a significant increase in P_4_ levels (39).

Correlation analysis revealed that EIR in the control group was significantly correlated with luteal characteristics at PD, whereas this relationship was absent in the nano–OVS group. Despite these different patterns, both groups showed positive correlations between CL number and diameter on day 27 post-AI, and between FCR and CL number, highlighting the importance of luteal function for successful pregnancy establishment. Larger or more numerous CL are typically associated with higher P_4_ secretion, which is essential for maintaining early pregnancy (65, 66).

The enhanced ovarian dynamics observed with nano–GnRH-BA were associated with differences in reproductive performance. Although FCR was numerically higher in nano–OVS-treated heifers, this difference was not statistically significant. In contrast, PL was significantly lower in heifers subjected to nano–OVS.

Both treatment groups showed distinct correlation patterns between ovarian activity on day 0 and fertility outcomes, supporting the differences in the physiological observations. In the standard OVS group, neither the presence of DF nor of CL at baseline (day 0) showed a significant correlation with FCR. The inherent variability in the ovulatory response to the first GnRH administration of the OVS protocol may explain this pattern. Previous studies have reported that ovulation in response to the first GnRH administration depends on the presence of a DF, and only ~50%−60% of females successfully ovulate following this administration, which can lead to incomplete follicular synchronization and reduced fertility outcomes (7, 67, 68). Additionally, variability in follicular dynamics and luteal regression can affect the efficiency of synchronization protocols when females are not subjected to presynchronization strategies (68, 69), thereby reducing the correlation between ovarian activity on day 0 and FCR.

In contrast, the nano–OVS group exhibited significant associations between ovarian structure on day 0 and FCR, indicating that the presence of DF and CL at the beginning of the protocol had greater predictive value for pregnancy in heifers subjected to nano–OVS. This finding may suggest that the inclusion of nano–GnRH-BA in the nano–OVS protocol enhances ovarian structural responsiveness to hormonal stimulation and improves the efficiency of synchronization between follicular development and ovulation. This improved synchronization may have contributed to the favorable reproductive performance outcomes obtained in heifers subjected to nano–OVS.

Overall, our observations suggest that incorporating a reduced dose of nano–GnRH-BA into the OVS protocol improves ovarian activity and follicular development, enhances synchronization efficiency, and promotes stronger luteal development after ovulation. These combined effects resulted in improved estrous induction, reduced PL, and enhanced reproductive performance in dairy heifers.

### 4.1 Limitations and future perspectives

Several limitations should be considered in the present study, including that treatment allocation was influenced by the availability of eligible heifers under practical field conditions, resulting in unequal group sizes. Moreover, the omission of hormonal profiles (LH, E_2_, and P_4_) would have provided additional insights into the endocrine mechanisms underlying the observed improvements in follicular development and luteal function. Additionally, this study primarily focused on ovarian structures detected by ultrasonography; therefore, molecular or cellular mechanisms associated with treatments were not evaluated.

Consequently, further research should investigate the endocrine and molecular profiles during the synchronization protocols. Further studies evaluating the precise pharmacokinetics of nanofabricated GnRH may help clarify the mechanisms by which nanodrug delivery systems enhance hormonal bioavailability. Moreover, studies involving larger populations and multiple reproductive outcomes would also be important to confirm the potential advantages of nano–OVS protocols and their applicability for improving reproductive performance in dairy herds.

## 5 Conclusion

Collectively, these findings demonstrate that incorporating GnRH-loaded CH nanocarriers into the OVS protocol was correlated with enhanced follicular and luteal development, improved EIR, and reduced PL in heifers. Although the nano–OVS–treated heifers exhibited a numerically higher FCR, this difference was not statistically significant. The observed results may be consistent with the proposed advantages of CH-NPs-based delivery systems; however, the stability, bioavailability, and pharmacokinetics dynamics of nano–GnRH-BA, as well as the direct endocrine responses, were not assessed in the current study. Therefore, the physiological and biological mechanisms underlying these effects remain to be confirmed.

This study, to our knowledge, is the first to integrate nano–GnRH-BA into a full OVS protocol in dairy heifers. Our observations suggest that the nano–OVS protocol may improve fertility outcomes, along with reducing hormonal dosage, offering a novel and efficient strategy for reproductive management in dairy heifers. These results highlight the potential of nanotechnology in precision reproductive management, providing a pathway to improve biological efficacy in livestock fertility programs.

Future research incorporating pharmacokinetic analyses, endocrine responses, and a larger animal population is required to confirm the translational potential of this approach and to facilitate its integration into different synchronization protocols to maximize reproductive performance.

## Data Availability

The raw data supporting the conclusions of this article will be made available by the authors, without undue reservation.
